# Drug repositioning *via* host-pathogen protein-protein interactions for the treatment of cervical cancer

**DOI:** 10.3389/fonc.2023.1096081

**Published:** 2023-01-25

**Authors:** Medi Kori, Beste Turanli, Kazim Yalcin Arga

**Affiliations:** ^1^ Department of Bioengineering, Faculty of Engineering, Marmara University, Istanbul, Türkiye; ^2^ Genetic and Metabolic Diseases Research and Investigation Center (GEMHAM), Marmara University, Istanbul, Türkiye

**Keywords:** host-oriented drugs, host-pathogen protein-protein interactions, cervical cancer, HPV, drug repurposing

## Abstract

**Introduction:**

Integrating interaction data with biological knowledge can be a critical approach for drug development or drug repurposing. In this context, host-pathogen-protein-protein interaction (HP-PPI) networks are useful instrument to uncover the phenomena underlying therapeutic effects in infectious diseases, including cervical cancer, which is almost exclusively due to human papillomavirus (HPV) infections. Cervical cancer is one of the second leading causes of death, and HPV16 and HPV18 are the most common subtypes worldwide. Given the limitations of traditionally used virus-directed drug therapies for infectious diseases and, at the same time, recent cancer statistics for cervical cancer cases, the need for innovative treatments becomes clear.

**Methods:**

Accordingly, in this study, we emphasize the potential of host proteins as drug targets and identify promising host protein candidates for cervical cancer by considering potential differences between HPV subtypes (i.e., HPV16 and HPV18) within a novel bioinformatics framework that we have developed. Subsequently, subtype-specific HP-PPI networks were constructed to obtain host proteins. Using this framework, we next selected biologically significant host proteins. Using these prominent host proteins, we performed drug repurposing analysis. Finally, by following our framework we identify the most promising host-oriented drug candidates for cervical cancer.

**Results:**

As a result of this framework, we discovered both previously associated and novel drug candidates, including interferon alfacon-1, pimecrolimus, and hyaluronan specifically for HPV16 and HPV18 subtypes, respectively.

**Discussion:**

Consequently, with this study, we have provided valuable data for further experimental and clinical efforts and presented a novel bioinformatics framework that can be applied to any infectious disease.

## 1 Introduction

Cervical cancer is the fourth most common cancer in women, according to the World Health Organization (WHO), and was responsible for 342,000 deaths worldwide in 2020 (1). It was estimated to be the second leading cause of death, especially among women in their 20s and 30s, showing the bitter truth (2). In addition, WHO has launched a global initiative to eliminate cervical cancer as a public health problem by 2020 (3). The main cause of cervical cancer is infection with the highly oncogenic human papillomavirus (HPV). In fact, more than 99% of cervical cancer patients are positive for one or more highly oncogenic HPVs. Among the 12 highly oncogenic HPV types, HPV-16 and -18 are the most prevalent subtypes worldwide and are responsible for up to 70% of cervical cancer cases (4). Because HPV is primarily a precursor for the development of cervical cancer, any effort to help understand the oncogenic impact of HPV or prioritize HPV-based innovative treatment strategies (i.e., host-oriented drug targets) will transform and advance bioscience research and the bioscience industry.

Thanks to biological networks that allow researchers to develop treatment strategies for complex diseases. Biological molecules never function alone; rather, they work together in a complex, interconnected network to carry out biological functions (5). These interplay between biological molecules also play a role in the development of diseases. Therefore, the construction and analysis of biological networks may be a favorable strategy to uncover the molecular mechanisms of disease and discover drugs that target and/or regulate the interactions between biological molecules (6). Nowadays, many studies reconstruct biological networks to explore the mechanisms behind diseases and discover drug candidates, especially for cancer (7–9).

Although various types of biological network models can be constructed to model the system, host-pathogen-protein-protein interactions (HP-PPIs) play a vital role in infectious diseases, because in infectious diseases, proteins of the pathogenic organism essentially interact with proteins of the host organism to influence their functionality (10). For this reason, host factors are critical to the survival of a pathogen. Therefore, drugs targeting viral proteins or host proteins that interact with the virus would be an effective strategy for developing efficient drug therapies, especially for infectious diseases (11). Since cervical cancer is an infectious disease of the cervix, HP-PPIs can provide remarkable data on the onset of oncogenesis and provide information for the development of effective treatment strategies. In one of the recent studies, the protein interaction maps of 12 HPV pathogens were constructed to find potential targets for drug development (12).

**Figure 1:**
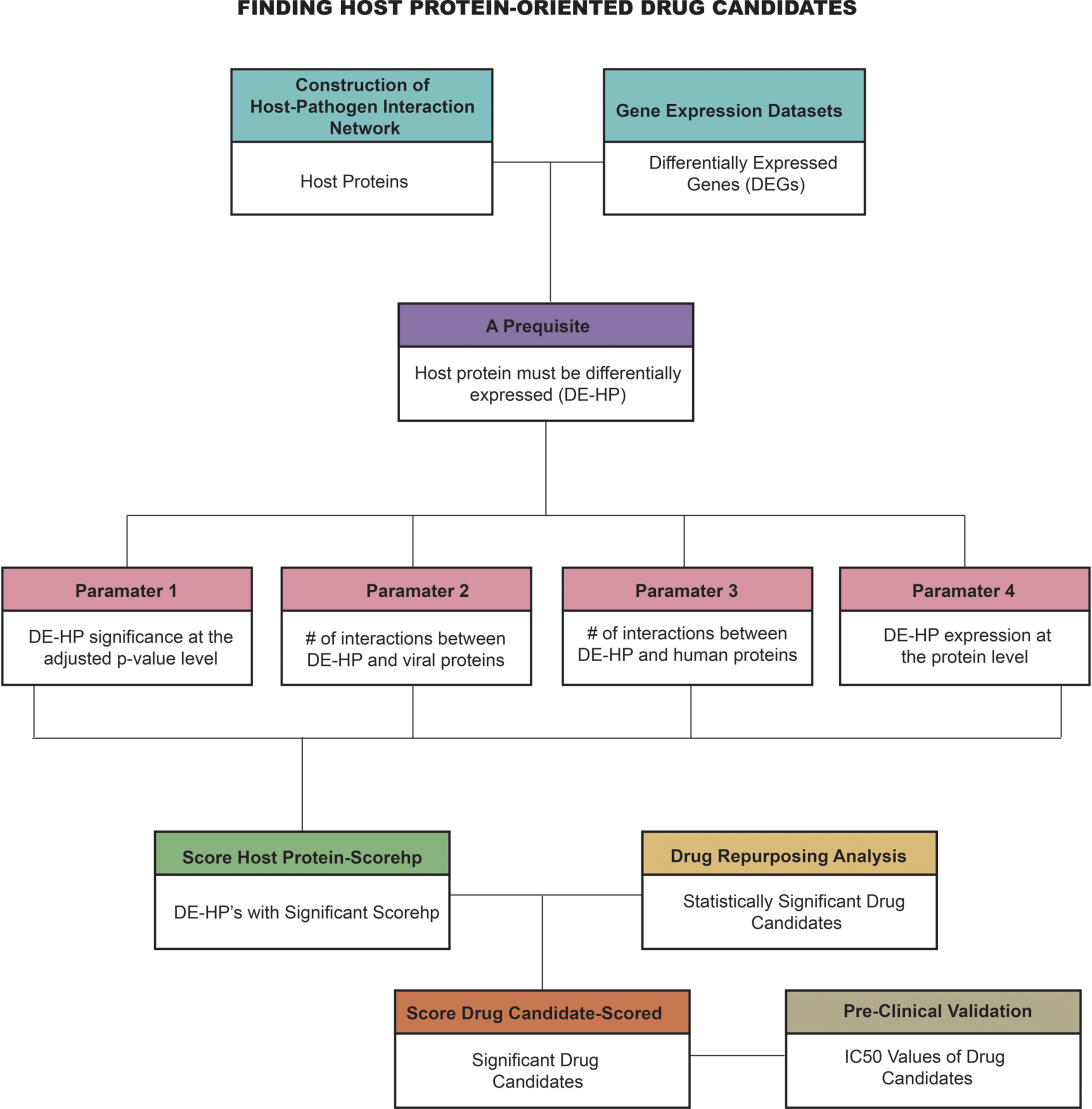
The novel computational framework employed in the study. (#, number).

In this study, given the importance of HP-PPIs and the potential differences between the HPV types most prevalent in cervical cancer (i.e., HPV16 and HPV18), a novel systems biology pipeline was used to develop efficient host-specific drug candidates against cervical cancer. The starting point of the study was the recognition that host proteins are an effective drug candidate for infectious diseases. However, we believe that each host protein has different properties and it cannot be assumed that every host protein is an effective drug target in every case. Therefore, we hypothesized that a host protein should stand out from the others based on its biological properties. For this reason, we set a prerequisite and four parameters separately to reveal their potential as drug targets. By integrating these four parameters, we scored the most suitable host proteins that can serve as effective drug targets (score host protein-scorehp). To find drug candidates, we performed drug repurposing analysis using prominent host proteins as drug targets (i.e., targets with significant scorehp). We then identified the most potentially effective drug candidates using the scoring approach that we developed (score drug-scored). Finally, we pre-clinically validated the highlighted drug candidates by evaluating half-maximal inhibitory concentration (IC50) values (**Figure 1**). Hereby, in this study, we identify novel framework that can be easily adapted for all infectious diaseases and discovered host-oriented drug candidates for cervical cancer based on host-pathogen interaction networks by specifying the potential differences between HPV16 and HPV18 subtypes.

## 2 Materials and methods

### 2.1 Data extraction: Host-pathogen protein-protein interactions

To collect the HPV16- and HPV18-associated host proteins, the HP-PPIs of both strains were taken from two publicly available biological repositories that collect experimentally verified HP-PPIs: Host-Pathogen Interaction Database (HPIDB v.3.0) (13) and Pathogen-Host Interaction Search Tool (PHISTO) (14). HP-PPI networks for each of the two HPV strains were reconstructed and represented as undirected graphs, with nodes representing host proteins and edges representing interactions between the virus and host proteins. The graphs were visualized using Cytoscape software (v.3.5.0) (15).

### 2.2 Evaluation of host proteins according to their biological features

After subtracting the HPV16- and HPV18-associated host proteins from the reconstituted HP-PPIs, we believe that certain host proteins should stand out from the others based on their biological properties. Therefore, we established a prerequisite and four parameters to specify the host proteins separately.

#### 2.2.1 A prerequisite: A host protein must be differentially expressed

We hypothesized that an effective host protein must be differentially expressed as a drug target. Differentially expressed genes (DEGs) are the genes whose expression levels are statistically different between different conditions. The fact that host proteins that have significant biological differences between the diseased and healthy states are valuable drug candidates because they are directly related to the disease and the use of these drugs is likely to increase the stability of treatment.

To this end, we evaluated the transcriptomic datasets considering the genotypes (i.e., HPV16 or HPV18) of the diseased samples using Gene Expression Omnibus (GEO) (16) to identify DEGs associated with cervical cancer. With this in mind, we found a total of five transcriptome datasets, namely GSE52903 (17), GSE39001 (18), GSE9750 (19), GSE7803 (20), and GSE6791 (21). While all five datasets contained HPV16 genotype samples, only three datasets (GSE9750, GSE7803, and GSE6791) contained HPV18 samples. Thus, a total of 111 HPV16-positive diseased samples were compared with 61 controls, while 10 HPV18-positive diseased samples were compared with 39 control samples.

To identify DEGs, raw data were read into the statistical software R using the Affy package (22) and normalized using Robust Multi-Array Average (23) implemented in the Bioconductor platform (version Rx64 4.0.2) (24). The normalized gene expression values were compared with the Linear Models for Microarray Data package (LIMMA) (25) to define DEGs. The Benjamini-Hochberg method was used as a control for false discovery rate. The adjusted p value < 0.05 was used as a cutoff value to determine the statistical significance of DEGs. Further analyzes were performed with the revealed DEGs that shared at least three of the five HPV16 transcriptome datasets, while analyzes were performed with the revealed DEGs that shared at least two of the three HPV18 transcriptome datasets to increase the robustness of the DEGs. In this way, the analysis continued with DEGs that were present in at least 60% of all datasets and were referred to as “core DEGs.”

To determine whether or not core-DEGs encode host proteins, we used GeneCards: The Human Gene Database (26). Host proteins that were differentially expressed were referred to as “differentially expressed host proteins (DE-HPs).”

#### 2.2.2 First parameter: Statistical significance of the association of host proteins with cervical cancer

As a first parameter, we count on the statistically significance of host proteins and cervical cancer relationship based on adjusted p-value level. Accordingly, the adjusted p-values of DE-HPs were reviewed individually for HPV16 and HPV18 datasets. Since there are different adjusted p-values from different datasets for DE-HPs, we considered the values that have the lowest adjusted p-value.

#### 2.2.3 Second parameter: The number of interactions between host and viral proteins

We hypothesized that the number of interactions between host and viral proteins is an important parameter for determining the prospering target protein. The application of a drug that targeting a host protein may also impress viral proteins. The higher the number of interactions between viral proteins and host proteins, means the more viral proteins will be affected. This increases the likelihood of preventing disease progression. Therefore, the number of interactions between DE-HPs and viral proteins is expected to be high for a successful target protein candidate. To determine the number of interactions between viral proteins and DE-HPs, the previously reconstructed HP-PPIs (13, 14) for HPV16 and HPV18 were examined and used.

#### 2.2.4 Third parameter: The number of interactions between host and human proteins

Similar to parameter two, if the drug target interacts with human proteins, the drug that affects the target will also affect the human protein through the interaction between them. Therefore, a possible effect of the drug on the target may have undesirable side effects on the human proteins, i.e., on the patient. Therefore, it is desirable that the number of interactions between the host protein and the human proteins is low so as not to affect the human proteins. To determine the number of interactions between DE-HPs and human proteins, DE-HPs were integrated with the human protein interactome. The human protein interactome was derived from a previously published study (27) containing 243,603 experimentally confirmed PPIs between 16,677 unique proteins from five data sources. When DE-HP does not interact with human proteins, the number of interactions is accepted as “1”.

#### 2.2.5 Fourth parameter: Host protein expression level in cervical cancer

We also evaluated the DE-HPs expression at the protein level. We thought high protein expression level in the disease state suggests that it plays an important role in disease development or prognosis and could likely be a notable drug target. To obtain DE-HPs expression at the proteome level, we used the Human Protein Atlas (HPA) (28). The HPA contains immunohistochemical staining profiles for proteins found in cancer tissues, including cervical cancer. The proteins are annotated for different staining levels as follows: high, medium, low, and not detected in the database. To express this situation numerically, the coefficients 3, 2, 1, and -1 were used for high, medium, low, and not detected, respectively, and multiplied accordingly. The calculated scores for a DE-HPs were then summed. Therefore, an “HPA score” was assigned for each DE-HPs found in the Atlas.

### 2.3 Integration of the specified parameters to determine of host protein score

We integrated the specified four parameter scores and assigned a final score for each DE-HPs to determine possibly most biologically prominent drug target (scorehp). A formula that we used to integrate the four parameters can be found in Equation 1 (Eq.1). The DE-HPs that indicated a positive scorehp were considered as “significant DE-HPs” and used for drug repurposing analysis.


(1) $$scorehp=-\text{log}[\text{min}.\left(\text{adjusted p}-\text{value}\right)\times \frac{\text{number of viral proteins interacting with host protein }}{\text{max}.\left(\text{number of human proteins interacting with host protein},1\right)}\times HPA\;score$$

### 2.4 Drug repurposing analysis

A web-based, transcriptome-driven drug repositioning tool, geneXpharma (29), was used to define drug candidates. Consequently, this tool provides the association of drug and disease (dataset) considering the hypergeometric distribution function. In this study, the gene-disease library was constructed using the detected core-DEGs of HPV16 and HPV18. By implementing the tool, we identified the drugs interacting with significant DE-HPs. Drugs with a hypergeometric p-value < 0.01 were considered statistically significant.

### 2.5 Determination of potential drug candidates

To obtain a promising match between drug and DE-HP, we integrated the scorehp and hypergeometric p-value of the drug to identify remarkable drug candidate. The formula we used to integrate the scorehp and hypergeometric p-value of the drug can be found in Equation 2 (Eq.2). This score was called the “drug score (scoredr)”. As a result of this scoring, the top 20% of scores are selected as potential drug candidates in rank order from highest to lowest.


(2) scoredr= −log[min. (hypergeometric p−value)]×scorehp

### 2.6 Pre-clinical validation of candidate drugs

To evaluate the efficacy of the revealed drug candidates, we used The Genomics of Drug Sensitivity in Cancer (GDSC) database (30), which is one of the largest public resources for information on the IC50 in cancer cells. The IC50 is a measure of a drug’s effectiveness in inhibiting a biological function and is widely used in pharmacology to evaluate drug efficacy. In this study, IC50 values of drug candidates in the cervical cancer cell lines SiHa (cell line with integrated HPV16 genome) and HeLa (cell line with integrated HPV18 genome) were used to evaluate the efficacy of potential drug candidates. To ensure consistency in our analyzes of drug efficacy, FDA-approved cervical cancer drugs were screened and considered positive controls.

## 3 Results

### 3.1 Reconstruction of virus-host protein networks to reveal host proteins

**Figure 2:**
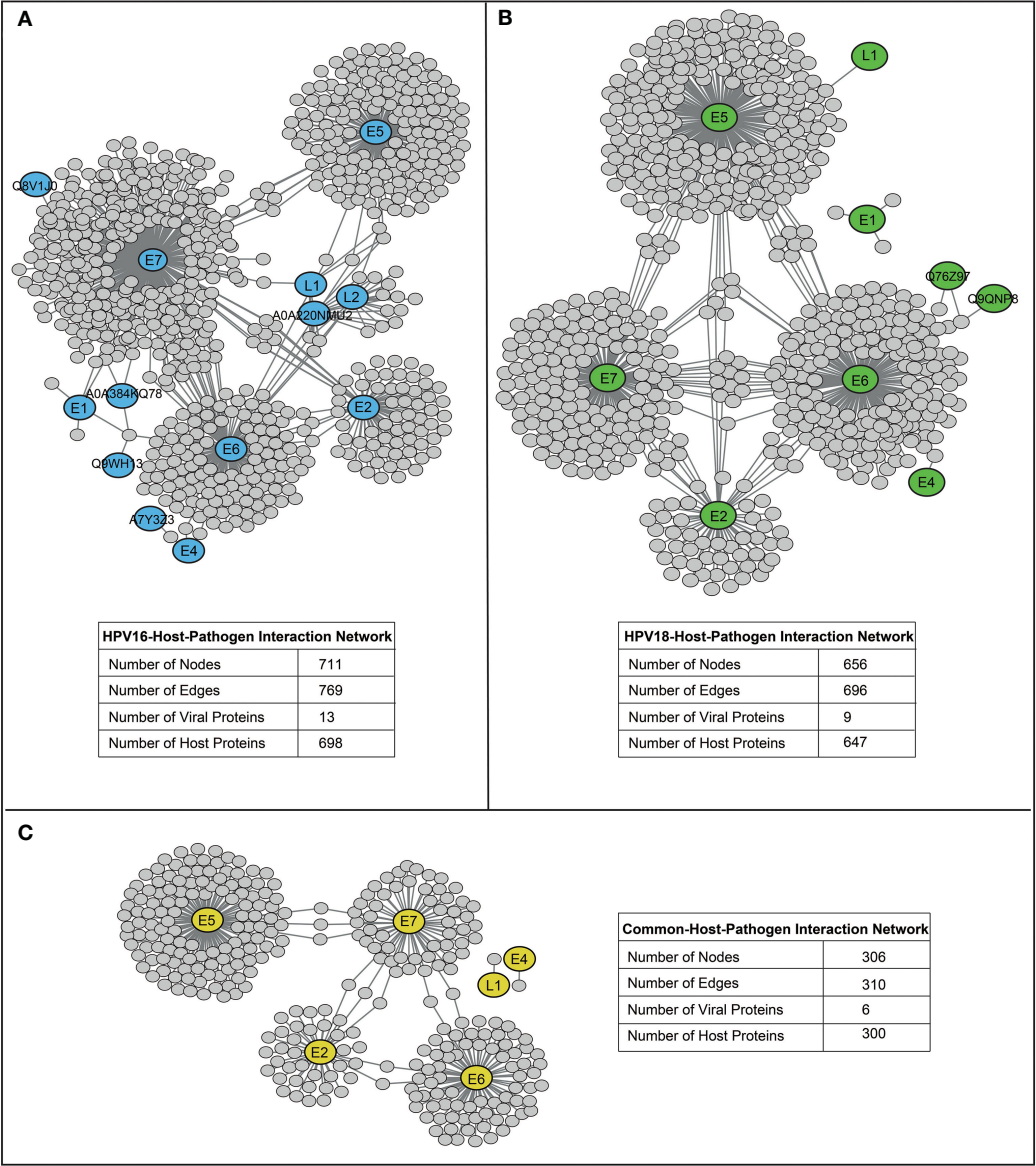
The reconstructed host-pathogen-protein interaction networks. **(A)** The reconstructed host-pathogen-protein interaction network for HPV16. **(B)** The reconstructed host-pathogen-protein interaction network for HPV18. **(C)** The host-pathogen-protein interaction network composed of mutual viral and host proteins of HPV16 and HPV18 strains.

HP-PPIs were obtained from two publicly available repositories (13, 14) to detect HPV16- and HPV18-associated host proteins. For HPV16, a total of 769 HP-PPIs consisting of 13 different viral proteins and 698 different host proteins were detected (**Figure 2A**), and for HPV18, a total of 696 HP-PPIs consisting of 9 different viral proteins and 647 different host proteins were detected (**Figure 2B**). Comparative analysis of the two networks revealed that the six viral proteins were common to both HPVs. Of these common seven viral proteins, six were encoded by the early expressed region (E2, E4, E5, E6, and E7), while one was encoded by the late expressed region known as L1. In addition, 300 host proteins were common to both HPVs (**Figure 2C**). We extracted the host proteins for both subtypes individually and screened them using the prerequisite and four parameters we established.

### 3.2 A prerequisite: If the host protein differentially expressed?

**Figure 3:**
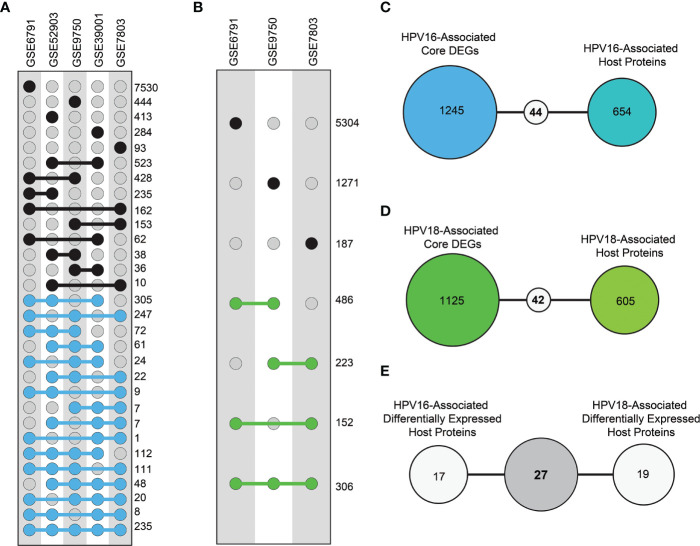
The distribution of differentially expressed genes (DEGs) and differentially expressed host proteins (DE-HPs). **(A)** The graph represents DEGs in transcriptome datasets with HPV16 samples from cervical cancer. The blue dots represent DEGs that share at least three of the five HPV16 datasets (i.e., core DEGs of HPV16). **(B)** The graph represents DEGs in the transcriptome datasets that involve HPV18 cervical cancer samples. The green dots represent DEGs present in at least two of the three HPV18 datasets (i.e., core DEGs of HPV18). **(C)** The diagram shows the number of common elements between HPV16-associated core DEGs and HPV16-associated host proteins. **(D)** The diagram shows the number of common elements between HPV18-associated core DEGs and HPV18-associated host proteins **(E)** The diagram shows the number of common host proteins between HPV16-associated DE-HPs and HPV18-associated DE-HPs.

As a prerequisite, we determined whether host proteins were encoded as differentially expressed. Therefore, we first determined DEGs using different transcriptome datasets. In this way, thousands of individual DEGs were found according to the criteria we established (i.e. adjusted p-value < 0.05). The resulting DEGs were analyzed comparatively. Accordingly, DEGs of HPV16 genotype were referred to as “HPV16 core-DEGs” if the DEGs occurred in at least three of five data sets. Similarly, DEGs of the HPV18 genotype were referred to as “HPV18 core-DEGs” if the DEGs were found in at least two of three data sets. In this way, a total of 1289 HPV16 core-DEGs (**Figure 3A**) and 1167 HPV18 core-DEGs were identified (**Figure 3B**). When we comparatively examined the revealed core-DEGs, we found that 562 of the core-DEGs were common to both subtypes (**Supplementary Table 1**).

We integrated the encoded core-DEGs with host proteins and found that 44 HPV16-associated (**Figure 3C**) and 46 HPV18-associated DE-HPs (**Figure 3D**). When DE-HPs were comparatively evaluated, 27 DE-HPs were found to be common to both subtypes (**Figure 3E**).

### 3.3 First parameter: What is the statistical significance of the differentially expressed host proteins in cervical cancer?

**Figure 4:**
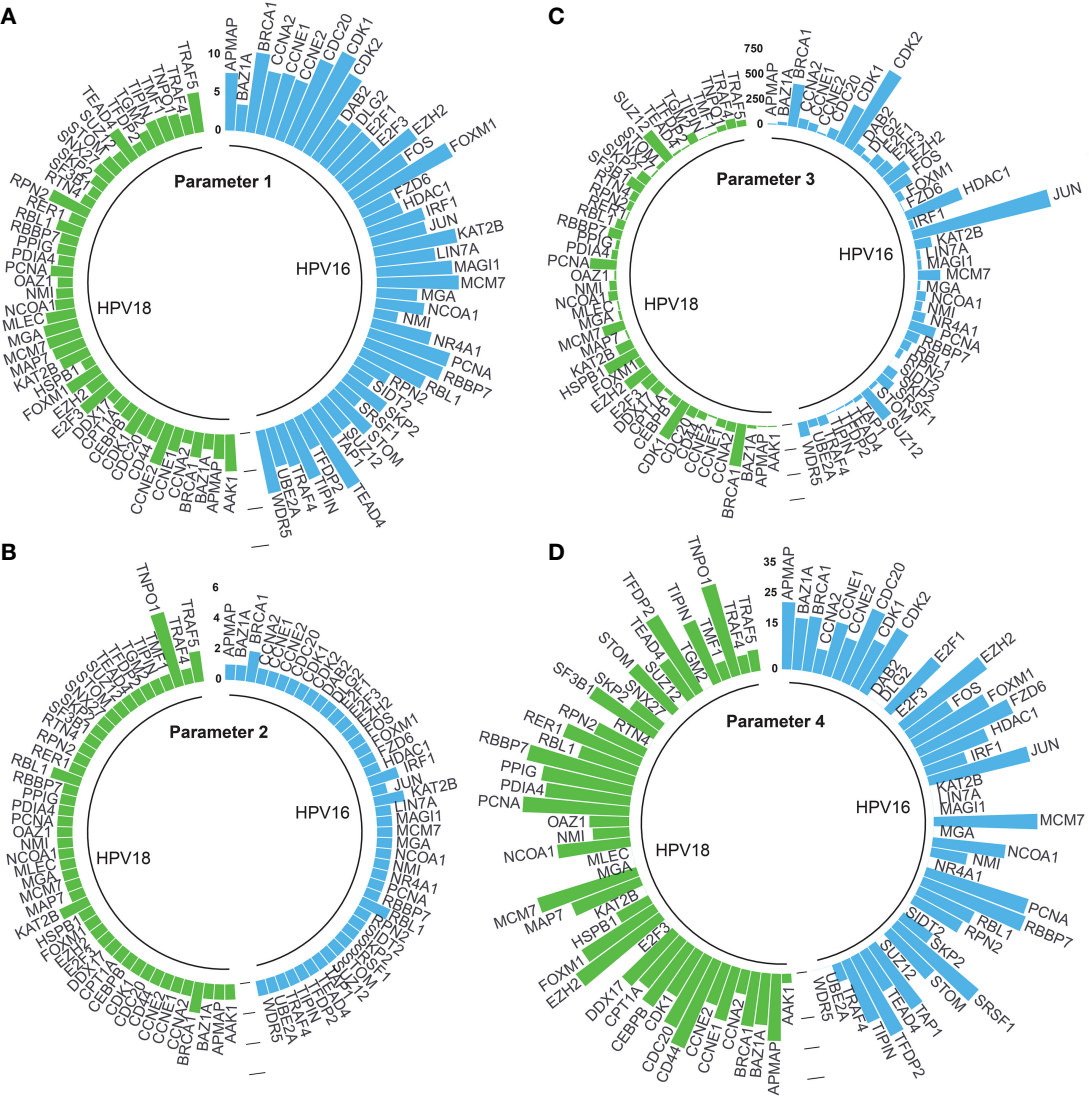
The circular bar graphs represent the results of the parameters determined in the study. **(A)** Parameter 1. The bar graph shows the values of differentially expressed host proteins (DE-HPs) at the adjusted p-value level (-log_10_). **(B)** Parameter 2. The bar graph shows the number of interactions between DE-HPs and viral proteins. **(C)** Parameter 3. The bar graph shows the number of interactions between DE -HPs and human proteins. **(D)** Parameter 4. The bar graph shows the calculated HPA scores for the DE-HPs. The negative HPA scores were represented as “0”.

Adjusted p-values of 44 and 46 HPV16- and HPV18-associated DE-HPs were evaluated to determine the statistical significance of DE-HPs in cervical cancer at the adjusted p-value level. Adjusted p-values for HPV16-associated DE-HPs ranged from 5.47×10^-15^ to 7.27×10^-4^, while adjusted p-values for HPV18-associated DE-HPs ranged from 2.72×10^-6^ to 3.56×10^-2^ (**Figure 4A**).

### 3.4 Second parameter: What is the number of interactions between host and viral proteins?

We expected that the number of interactions between DE-HPs and viral proteins should be high. To reveal interactions between them, we used the obtained HP-PPIs (13, 14). HPV16-associated DE-HPs (i.e., BRCA1, IRF1, KAT2B, and RBL1) had at most two interactions with HPV16 viral proteins. One of the HPV18-associated DE-HP, TNPO1, had five interactions with viral proteins. While the other HPV18-associated DE-HPs had two or one interaction with HPV18 viral proteins (**Figure 4B**).

### 3.5 Third parameter: What is the number of interactions between host and human proteins?

We assumed that the number of interactions between DE-HPs and human proteins should be few. To reveal interactions between them, we used the human protein interactome (27). An HPV16-associated DE-HP, JUN, has the highest number of interactions with human proteins (1405 interactions), whereas SIDT2, has no interaction with human proteins. An HPV18-associated DE-HP, BRCA1, has the highest number of interactions with human proteins (423 interactions). The two HPV18-associated DE-HPs, APMAP and TIPIN, have the fewest interactions with human proteins (nine interactions) (**Figure 4C**).

### 3.6 Fourth parameter: What is the host protein expression level in cervical cancer?

We investigated the protein expression of DE-HPs in cervical cancer. To determine protein expression of DE-HPs, we used the HPA database (28) and assigned an HPA score to each DE-HP as described in the Materials and Methods section. HPA scores for both HPV16- and HPV18-associated DE-HPs ranged from 36 to (-) 12 (**Figure 4D**).

### 3.7 Integration of parameters to determine most optimal host protein target

**Figure 5:**
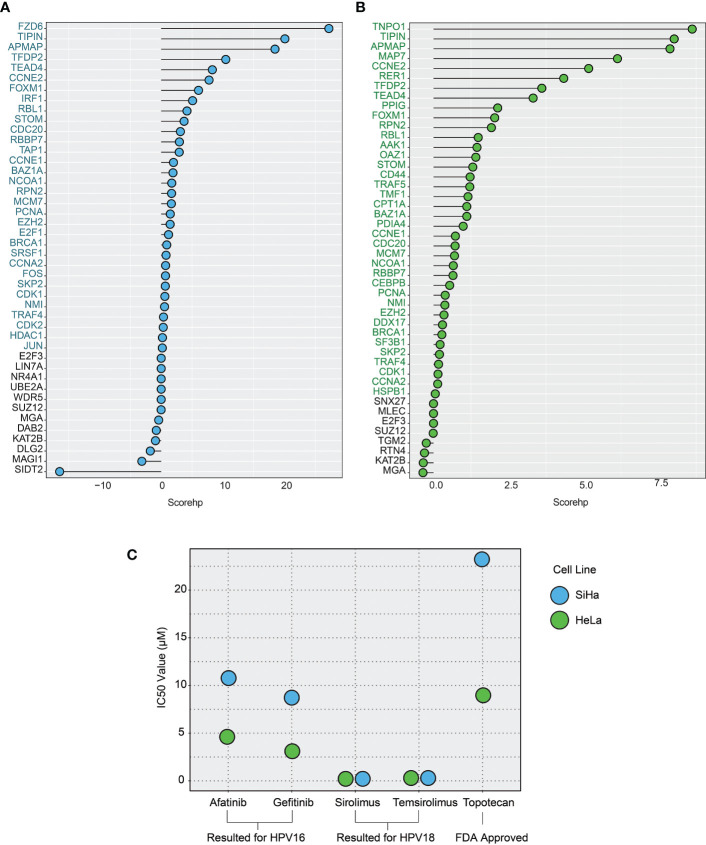
The plots show the host protein score (scorehp) results for differentially expressed host proteins (DE-HPs) and the IC50 values of the drug candidates found. **(A)** A plot of HPV16-associated DE-HP’ scorehp results. The DE-HPs in blue were considered significant based on their scorehp results in the study. **(B)** A plot of HPV18-associated DE-HP’ scorehp results. The DE-HPs in green were considered significant based on their scorehp results in the study. **(C)** The IC50 values (µM) of the drug candidates for the SiHa and HeLa cell lines. Only the significant results were shown.

By integrating the above four parameters, we calculated the scorehp for each DE-HPs of HPV16 and HPV18. The DE-HPs that showed a positive scorehp were considered significant and used as targets for drug repurposing analysis. Accordingly, we determined that 4.5% of the total host proteins of HPV16 (32 DE-HPs) (**Figure 5A**) and 5.8% of the total host proteins of HPV18 (38 DE -HPs) (**Figure 5B**) would be effective host target proteins.

### 3.8 Drug repurposing analysis and determination of host protein drug score

A drug repositioning tool, geneXpharma (29), was used to define drug candidates that target significant DE-HPs (i.e., have positive a scorehp). A total of 53 different significant drugs were found targeting 32 HPV16-associated DE-HPs (**Supplementary Table 2**), while a total of 37 different significant drugs were found targeting 38 HPV18-associated DE-HPs (hypergeometric p-value < 0.01) (**Supplementary Table 3**).

To obtain a more promising match between statistically significant drug candidates and DE-HPs, the determined scorehp and hypergeometric p-value scores were integrated to obtain scoredr. According to the results of scored, 19 different drug candidates for HPV16-associated DE-HPs and 11 different drug candidates for HPV18-associated DE-HPs were identified (**Table 1**). Of these drugs, 7, namely BAY1000394, cetuximab, erlotinib, lapatinib, letrozole, panitumumab, and trastuzumab, were identified for both subtypes. A total of 12 drugs (afatinib, canertinib, everolimus, gefitinib, interferon alfacon-1, lidocaine, neratinib, perifosine, pimecrolimus, ridaforolimus, saracatinib, and temsirolimus) were identified specifically for the HPV16 subtype. The 4 drugs (asparaginase, hyaluronan, mifepristone, and sirolimus) were found to be specific for the HPV18 subtype.

**Table 1:** The candidate drugs association with cervical cancer.

DRUG	ASSOCIATION WITH CERVICAL CANCER	REFERENCE
***HPV16-SPECIFIC DRUG CANDIDATES***		
Afatinib	EGFR-amplified metastatic cervical cancer patient benefiting from afatinib as a single agent	(31)
Canertinib	Canertinib combined with Pd(II) complex leads to inhibition of migration and invasion.	(32)
Everolimus	Combining everolimus, cisplatin and pelvic radiotherapy for locally advanced cervical cancer is therapeutic strategy	(33)
Gefitinib	Gefitinib may be a potential novel therapeutic strategy in cervical cancer by suppressing the Wnt/β-catenin signaling pathway and EMT to inhibit tumor metastasis in cervical cancer cells.	(34)
Interferon Alfacon-1	–	Novel
Lidocaine	Lidocaine repressed the growth of cervical cancer cells by modulating the lncRNA-MEG3/miR-421/BTG1 pathway	(35)
Neratinib	Neratinib monotherapy showed evidence of activity in heavily pretreated patients with HER2-mutant cervical cancer	(36)
Perifosine	Perifosine monotherapy showed good tolerability in patients with ovarian, endometrial, or cervical cancer.	(37)
Pimecrolimus	–	Novel
Ridaforolimus	Treatment with ridaforolimus in combination with paclitaxel and carboplatin showed antineoplastic activity in tumors including cervical cancer	(38)
Saracatinib	Saracatinib inhibits Src activation, invasion and cervical lymph node metastasis in an orthotopic mouse model of oral squamous cell carcinoma	(39)
Temsirolimus	Single agent temsirolimus has modest activity in cervical carcinoma	(40)
***HPV18-SPECIFIC DRUG CANDIDATES***		
Asparaginase	L-asparaginase can effectively inhibit the growth of human cervical cancer cells	(41)
Hyaluronan	–	Novel
Mifepristone	Mifepristone inhibits the migration of cervical cancer cells by inhibiting exocrine secretion	(42)
Sirolimus	Sirolimus may inhibit hypoxic HeLa cell proliferation through the trigger of programmed cell death	(43)
Cetuximab	NCT00101192	(44)
***DRUG CANDIDATES THAT COMMON IN BOTH SUBTYPES***		
Panitumumab	NCT01158248	(44)
Bay1000394	Shows antitumor activity in athymic mice bearing established HeLa-MaTu cervical xenograft tumors.	(45)
Erlotinib	Erlotinib combined with cisplatin-based chemoradiation exerts significant activity against locally advanced cervical cancer.	(46)
Lapatinib	Pazopanib and lapatinib both demonstrated a favorable toxicity profile in patients with advanced and recurrent cervical cancer.	(47)
Letrozole	NCT02482740	(44)
Trastuzumab	The combination of trastuzumab-pertuzumab, and the single-agent TDM-1, being effective in treatment of cervical cancer with HER2 amplification	(48)

To determine whether these 23 candidate drugs had been previously associated with cervical cancer, we conducted an extensive literature search. We found that many of the drugs discovered had been used in or associated with cervical cancer (31–48) (**Table 1**). Because studies in the literature generally do not include information on the HPV genotype of patients, we reviewed the drugs without considering HPV genotype to be on the safe side, and the drugs that had ever been associated with cervical cancer were not considered new in this study. In addition, to our knowledge, interferon alfacon-1, pimecrolimus (especially for HPV-16-infected patients), and hyaluronan (especially for HPV-18-infected patients) were first introduced as drug candidates for the treatment of cervical cancer in our study.

### 3.9 The pre-clinical validation of efficiencies of potential drug candidates

To evaluate the efficacy of the discovered drug candidates, we evaluate the IC50 values of the drugs. In addition, a drug, topotecan, which is FDA-approved for cervical cancer (49), was included in the validation analysis to compare the IC50 values. By the validation analysis, we want to evaluate if the IC50 value of an FDA-approved drug is higher than that of already associated drugs. Indeed, if it is high, it means that the already associated drug has the potential to be approved for routine use in the clinic, like topetecan. In addition, a high IC50 value for topetecan strengthens our confidence in our observations on the new drugs.

Among the 23 drugs already associated with cervical cancer, we found IC50 values of seven drugs. The results suggest that the drugs afatinib and gefitinib (which we found to be specific for the HPV16 subtype) have lower IC50 values compared with topotecan in SiHa cell lines. The IC50 values of sirolimus and temsirolimus, which we found to be specific for the HPV18 subtype, showed that these drugs were more efficient (lower IC50 values) than the use of topotecan in HeLa cells (**Figure 5C**).

## 4 Discussion

Biological networks are the root of the structure of living things. With biological networks, we can profoundly understand the organization of the whole organism. This allows us to understand the holistic mechanisms of the system and to exploit the enormous potential for drug development. In infectious diseases, including cervical cancer, analysis of the biological network comprising the virus-host interactome, which provides a comprehensive view of the interactions between a virus and its host protein, is a novel strategy for developing effective treatment strategies (50, 51). However, the analysis of virus-host interactomes is a field that stands on stony ground. Researchers are gradually beginning to recognize the importance of HP-PPIs and are starting to support this field, especially with the ongoing 2019 coronavirus pandemic (COVID -19) (52–54).

Drug therapies targeting host proteins offer great potential for the present and the future, especially for infectious diseases such as viral cancers. For human viruses, viral-targeted drug therapies have traditionally been used. However, it is now clear that virus-oriented drug therapies have several drawbacks. One of the main disadvantages is that there are few viral proteins that can be treated with drugs, and even when there are, the drug targets for viral proteins are extremely limited. In addition, the viral genome is rapidly evolving, which should be taken into account (11). Moreover, the emergence of resistance to viral targeted drug therapies is an inevitable end that can complicate the treatment of all diseases today (55). Because of these advantages, various efforts have been made recently to identify basic host factors as targets and develop host-oriented drugs.

In this study, given the limitations of virus-directed drug therapies, we focused on discovering potential host-directed drug candidates for cervical cancer. We believe that H -PPIs could be the starting point for the discovery of host agents. However, we believe that host proteins should be prioritized according to their biological functions. Therefore, we developed a novel framework for prioritizing host proteins. Instead of discovering drugs for prioritized host proteins from scratch, we performed drug repositioning analyzes to discover repositioned drugs that target host proteins, which is more advantageous than traditional drug discovery approaches. Drug repositioning analysis offers time and cost savings compared with traditional approaches to drug development (56). Accordingly, by integrating prioritized host proteins and host-targeted repositioned drugs as part of the novel framework, we have already presented associated and novel host-targeted drug candidates for cervical cancer.

Interferon alfacon-1, pimecrolimus (especially for HPV-16 infected patients) and hyaluronan (especially for HPV-18 infected patients) were first introduced as drug candidates for the treatment of cervical cancer with our study. Interferon alfacon-1 (consensus interferon) is an unnatural synthetic interferon-alpha type 1 used to treat patients with chronic hepatitis C (57). Pimecrolimus was developed specifically for the treatment of inflammatory skin diseases (58). Hyaluronan has been approved for the treatment of osteoarthritis. It has also been used in embryo implantation and wound healing (59). In addition to discovering novel drug candidates, this novel framework shows productive results when we evaluating the IC50 values of previously associated cervical cancer drug candidates. The host-targeted drugs afatinib (31), gefitinib (34), sirolimus (43), and temsirolimus (40) were found to be more effective than the FDA-approved drug for cervical cancer (i.e., topetecan) when their IC50 values were compared. Accordingly, these IC50 results have further strengthened our confidence in our observations and our developed system.

The major limitation of the study is the lack of experimental validation of the novel drugs with relevant tissue samples or cell lines. Future *in vitro* studies need to be performed to investigate and test the effects of the identified novel drugs in terms of their response to disease, cell viability, disease progression, and migration. In addition, the consistency, reproducibility, and reliability of the drugs presented in this study should be experimentally validated in patients to prove that the drugs will be clinically useful. We believe that computational analysis is an important and first step in drug development. However, to address a broad medical audience, the need for experimental validation is inevitable.

In conclusion, in this study, we focus on the discovery of host-specific drug candidates by investigating HP-PPIs to find efficient drug candidates for cervical cancer. With this study, we have developed a novel framework that will help us discover new and efficient host-oriented drugs. Although we used the developed framework for HPV16- and HPV18-associated cervical cancer cases, this framework can be easily adapted to achieve rapid data generation and can be used as a weapon to combat infectious diseases. Moreover, with this study, we have provided valuable data for further experimental and clinical efforts, as the proposed novel drug candidates are the potential therapeutic targets for the treatment of cervical cancer.

## Data availability statement

The original contributions presented in the study are included in the article/**Supplementary Material**. Further inquiries can be directed to the corresponding author.

## Author contributions

KA designed the study. MK performed the analyses and wrote the manuscript. MK, BT, and KA revised and contributed to the manuscript. All authors contributed to the article and approved the submitted version.
